# Transducer-Like Protein in *Campylobacter jejuni* With a Role in Mediating Chemotaxis to Iron and Phosphate

**DOI:** 10.3389/fmicb.2018.02674

**Published:** 2018-11-16

**Authors:** Kshipra Chandrashekhar, Vishal Srivastava, Sunyoung Hwang, Byeonghwa Jeon, Sangryeol Ryu, Gireesh Rajashekara

**Affiliations:** ^1^Food Animal Health Research Program, Department of Veterinary Preventive Medicine, The Ohio State University, Wooster, OH, United States; ^2^Department of Food and Animal Biotechnology – Department of Agricultural Biotechnology, Center for Agricultural Biomaterials, Seoul National University, Seoul, South Korea; ^3^School of Public Health, University of Alberta, Edmonton, AB, Canada

**Keywords:** transducer like protein, chemotaxis, iron, regulation, promoter

## Abstract

Chemotaxis-mediated motility enables *Campylobacter jejuni* to navigate through complex environmental gradients and colonize diverse niches. *C. jejuni* is known to possess several methyl accepting chemotaxis proteins (MCPs), also called transducer-like proteins (Tlps). While the role of some of the Tlps in chemotaxis has been identified, their regulation and role in virulence is still not very clear. Here, we investigated the contribution of Tlp2 to *C. jejuni* chemotaxis, stress survival and colonization of the chicken gastrointestinal tract. The Δ*tlp2* deletion mutant showed decreased chemotaxis toward aspartate, pyruvate, inorganic phosphate (Pi), and iron (FeSO_4_). Transcriptional analysis of *tlp2* with a promoter fusion reporter assay revealed that the *tlp2* promoter (P*_tlp2_*) was induced by Pi and iron, both in the ferrous (Fe^2+^) and ferric form (Fe^3+^). RT-PCR analysis using overlapping primers indicated that the *phoX* gene, located immediately downstream of *tlp2,* is co-transcribed with *tlp2*. A transcription start site was identified at 53 bp upstream of the *tlp2* start codon. The Δ*tlp2* mutant showed decreased colonization of the chicken gastrointestinal tract. Collectively, our findings revealed that the *tlp2* plays a role in *C. jejuni* pathogenesis and colonization in the chicken host and its expression is regulated by iron.

## Introduction

Foodborne gastrointestinal illness caused by a gram negative bacterium, *Campylobacter jejuni,* has seen a surge in incidence in the recent years (CDC, 2013). In the United States, Food and Drug Administration (FDA) has placed *Campylobacter* species in the list of “qualifying pathogens” capable of posing a serious public health risk (Food and Drug Administration and HHS, 2014). The prevalence and transmission of *Campylobacter* can be attributed to its widespread colonization in the gastrointestinal tract of farm animals, especially chickens (Hermans et al., 2012). It is well established that *C. jejuni* employs motility and chemotaxis to colonize the avian and mammalian gastrointestinal tract (Yao et al., 1994; Hendrixson and DiRita, 2004; Young et al., 2007; Hermans et al., 2011; Chandrashekhar et al., 2015, 2017). Directional motility in *C. jejuni* is mediated by the chemotaxis system, composed of chemoreceptors and other core signal transduction proteins (Lertsethtakarn et al., 2011).

Transducer like proteins (Tlps) are the key components involved in sensing environmental signals through chemotaxis or energy taxis in *C. jejuni*. (Marchant et al., 2002; Vegge et al., 2009; Korolik, 2010; Tareen et al., 2010; Reuter and van Vliet, 2013; Rahman et al., 2014). Amino acids (aspartate, glutamate and serine), organic acid salts (succinate, isocitrate, and formate), bile and mucin are chemoattractants for *C. jejuni* (Hugdahl et al., 1988; Hartley-Tassell et al., 2010; Tareen et al., 2010). *C. jejuni* Tlps have been classified into three groups (A-C), based on sequence analysis and structural homology (Marchant et al., 2002; Chandrashekhar et al., 2017). The *C. jejuni* Tlp2 (CJJ81176_0180) is a group A transducer-like protein (Marchant et al., 2002) with transmembrane domains, a periplasmic ligand binding domain and a cytoplasmic signaling domain. BLAST analysis of the predicted amino acid sequence of Tlp2 shows greatest homology to *C. jejuni* Tlp3 and Tlp4 (60% identity). The cytoplasmic signaling domain is identical to Tlp3 but the periplasmic domain shows only 38% identity with Tlp3 (Rahman et al., 2014). An earlier study in *C. jejuni* NCTC11168 strain revealed that *tlp2* deletion mutant exhibited no chemotaxis and invasion defects (Vegge et al., 2009). However, recent evidence indicates that *tlp2* is one of the most abundantly expressed *tlps* in mice infected with *C. jejuni* NCTC 11168-O (Day et al., 2012), thus emphasizing the significance of understanding the role of Tlp2 in *C. jejuni* pathophysiology. This warranted us to further investigate the role of *C. jejuni* Tlp2 in chemotaxis, virulence, and host colonization.

Iron is an essential nutrient and a cofactor for proteins involved in cellular metabolism, enzyme catalysis, and sensing extracellular and intracellular signals (Lill, 2009). The bioavailability of iron in the host and environment (10^−18^–10^−24^M) being lower than the minimum requirement for bacterial growth (10^−7^M), makes iron a key player in the host-pathogen interaction (Braun and Hantke, 2003). Chemotaxis toward iron has been studied in *Shewanella oneidensis* and the magnetotactic bacteria *Geobacter metallireducens* (Childers et al., 2002; Bencharit and Ward, 2005). In these bacteria, the chemotactic response to iron is due to the fact that it serves as an insoluble electron acceptor (Childers et al., 2002; Bencharit and Ward, 2005; Harris et al., 2010). Knowledge about the role of iron as an electron acceptor in *C. jejuni,* chemotaxis toward iron and/or regulation of Tlp genes by iron in *C. jejuni* is still scarce. However, a study in *Helicobacter pylori* indicated that *tlpB,* chemoreceptor for sensing bicarbonate and arginine, is induced by iron through a *fur-*independent mechanism (Ernst et al., 2005). Interestingly, a recent study in *C. jejuni* has identified that *tlp* genes (Cj0262c and Cj1110c) are regulated by iron and/or Ferric uptake regulator (Fur) protein (Butcher et al., 2012). The study also revealed that *cj0145* (*phoX*), a gene located immediately downstream of *tlp2,* is induced in the presence of iron although the specific mechanism of regulation is still unexplored (Butcher et al., 2012).

Here we investigated the role *tlp2* in *C. jejuni* chemotaxis, *in-vitro* virulence and colonization of the chicken gastrointestinal tract. We provide evidence that iron regulates chemotaxis in *C. jejuni* and *tlp2* contributes to *in-vivo* colonization of the chicken gastrointestinal tract. The findings of this study not only highlight the significance of *tlp2* in *C. jejuni* pathogenesis but also elaborate on the complex mechanism by which iron regulates the chemotaxis in *C. jejuni* through Tlp2.

## Materials and Methods

### Bacterial Strains, Media and Growth Conditions

Bacterial strains and plasmids used in this study are described in Table 1. *C. jejuni* used in this study are derivatives of strain 81–176 (WT) (Korlath et al., 1985) and NCTC 11168. *C. jejuni* strains were grown on Mueller-Hinton media (MH; Oxoid, Hampshire, United Kingdom) under microaerophilic conditions [(85% N_2_ (v/v), 10% CO_2_ (v/v) and 5% O_2_ (v/v)] in a DG250 Microaerophilic Workstation (Microbiology International, Frederick, Maryland, United States) at 42°C. *E. coli* DH5α was used for plasmid propagation and cloning purposes and was routinely cultured on Luria-Bertani (LB) medium at 37°C overnight. Growth media was supplemented with appropriate antibiotics; chloramphenicol (10 μg/ml for *Campylobacter*; 20 μg/ml for *E. coli*) and kanamycin (30 μg/ml for *Campylobacter*; 50 μg/ml for *E. coli*) as required.

**Table 1:** Bacterial strains and plasmids used in this study.

Strains	Relevant description	Source/Reference
*C. jejuni* 81–176 WT	Wild type strain of *C. jejuni*	Dr. Qijing Zhang
*Δtlp2*	*C. jejuni* 81–176 derivative with deletion in *tlp2* gene; *tlp2*::kan	This study
*Δtlp2-cm*	*C. jejuni* 81–176 derivative with deletion in *tlp2* gene; *tlp2*::cm	This study
*tlp2 comp*	*C. jejuni 81–176 tlp2* mutant complemented with wild type copy of *tlp2* on pRY112	This study
*C. jejuni* NCTC11168*Δfur*	*C. jejuni* NCTC11168 derivative with deletion in *fur* gene; *fur*::tet	Dr. Jun Lin
WT P*_tlp2_*-pMW10	*C. jejuni* 81–176 WT reporter strain carrying P*_tlp2_*-pMW10	This study
Δ*tlp2* P*_tlp2_*-pMW10	*C. jejuni* 81–176 Δ*tlp2* strain carrying P*_tlp2_*-pMW10	This study
Δ*fur* P*_tlp2_*-pMW10	*C. jejuni* 81–176 Δ*fur* strain carrying P*_tlp2_*-pMW10	This study
WT P*_int_*-pMW10	*C. jejuni* 81–176 WT reporter strain carrying *P_tlp2-phoXint_*-pMW10	This study
*E. coli* DH5α	*E. coli* strain used for cloning	Invitrogen
**Plasmids**		
pZero-1	Cloning vector for making suicide vector; Zeo	Invitrogen
pUC4K	Source plasmid for kanamycin resistance gene; Kan	Amersham
pUC4C	Source plasmid for chloramphenicol resistance gene; Cm	This study
pMW10	Promoter shuttle vector; pMW10, Kan	Wosten et al., 1998
pRY112	*E.coli*-Campylobacter shuttle vector for complementation; Cm	Yao et al., 1993
pRK2013	Helper plasmid for complementation; Kan	Grabowska et al., 2011
pZero1-*tlp2*	pZero-1 containing the upstream and downstream sequences of *tlp2*; Zeo	This study
pZero1-*Δtlp2*-kan	pZero1-*tlp2* with *tlp2* gene replaced by the pUC4K kan gene through inverse PCR; Zeo, Kan	This study
pZero1-*Δtlp2*-cm	pZero1-*tlp2*-kan where kanwas replaced by the chloramphenicol gene; Zeo, Cm	This study
P*_tlp2_*-pMW10	pMW10 carrying the *tlp2* promoter; Kan	This study
P*_int_*-pMW10	pMW10 carrying the intergenic region between *tlp2* and *phoX*; Kan	This study

*Cm, chloramphenicol resistance; Kan, kanamycin resistance; Zeo, Zeocin resistance.*

### Generation and Complementation of *tlp2* Mutant

Recombinant DNA techniques were performed as per standard procedures (Sambrook et al., 1989). *C. jejuni tlp2* mutant was created by double crossover allelic exchange method as previously described (Rajashekara et al., 2009). Oligonucleotides used in the present study were synthesized from Integrated DNA Technologies (Skokie, IL, United States) and are listed in Table 2. Briefly, the gene of interest (*tlp2*) plus ∼ 1 kb flanking DNA was amplified by PCR from *C. jejuni* strain 81–176 genome. The purified PCR products were ligated into zeocin-resistant pZErO-1 (zero background cloning vector) (Invitrogen, Carlsbad, CA, United States), and the ligation product was transformed into Library Efficiency DH5α *E*. *coli* competent cells (Invitrogen) to generate the plasmid pZErO1-*tlp2*. The whole plasmid except the target gene was amplified by inverse PCR. Purified inverse PCR products were ligated either to a kanamycin resistant cassette (from pUC4K) or a chloramphenicol resistance cassette (from pUC4C), and the resulting suicide vector was electroporated into *C*. *jejuni*. Transformants were selected on MH agar supplemented with chloramphenicol or kanamycin. Individual clones were confirmed for deletion of the target gene by PCR. The *tlp2* mutant with kanamycin resistance was used in all the assays; except for reporter studies, in which case *tlp2* mutant with chloramphenicol resistance was used as reporter plasmid carries kanamycin resistance.

**Table 2:** Oligonucleotide primers used in this study.

Name	Sequence
**Primers for gene deletion**	
*tlp 2*F	ATATATGGTACCTTGCTACTAGTAT TTTGTTC
*tlp 2*R	AATTAACTCGAGCATAACCTTGT GGTACTATA
*tlp 2*F inv	ATATATGGATCCGAGAACA TGGTAGAGGCTTT
*tlp 2*R inv	ATATATGGATCCCCAGCATC TCTAAAATTCTT
**Complementation primers**	
*tlp 2* comp F	AATGAAGTCGACAAATTATA ACGATATTAAGC
*tlp 2* comp R	AATTAAGGTACCAAAACCTTT TCTTCTTAACA
**Primers for RT-PCR and (q)RT-PCR**	
Intergenic region P-180/181 (P1) F	ATAGCGTAGCTCAAATTGAT
Intergenic region P-180/181 (P1) R	AAGCATAGCAGCACTTAAAT
*tlp2* control (P2) F	TGCAAATCTTGCTAAAACTA
*tlp2* control (P2) R	GTCCAAATTCATCATTGCTT
*phoX* control (P3) F	GCTATGGATTTAACAAAACT
*phoX* control (P3) R	GTTAAACTGTCCTACATACA
*phosR* F	GCAAACATAATCATCACAACCAC
*phosR* R	GAGAGCAAGGATACAAAGAAGC
*pstS* F	CCTTATACAAACTGGAATCAAATC
*pstS* R	GACACATCACTCATTACAAGC
*pstC* F	CGCTTATGCTTTAGGTATGAC
*pstC* R	GCTGCCATCACCACTATC
**Primers for promoter fusion reporter studies and primer extension assay**	
*tlp2*_PF_F	ACA TTG ACA TCC CGG GTA TTT GCA GC
*tlp2*_PF_R	AAT CAG TGA GAT CTT CAA TTT TAC GC
CJJ81-180_PE_temp_F	GGG GGC AAA ATA ACA TTG ACA TCT AGA G
CJJ81-180_PE_temp_R	GCA TCT TGA CTA TCT AAC TGT TCT ATA GG
CJ81-180_PE_R1	ACC TAA AAT TAT CAA ACA CAC TAC TGC G
CJJ81-180_PE_R2	TAA TTT ATT TCA GCA TTC ACA ACT TCA TG
CJJ81-181_PE_temp_F	AAA CTG CAG GTA TCA CTC AAA TCA ATG
CJJ81-181_PE_temp_R	ACC TAG CAA ATC CTT ATC CTT AAG C
CJJ81-181_PE_R1	TTG CAA AAA AAG CCA CCA TAG AAC C
CJJ81-181_PE_R2	TTA AAA CCT TTG CTT CAT AAC CTT GTG G

The complemented strain was created by amplifying coding regions of *tlp2* along with its potential promoter region by PCR using primers indicated in Table 2. The resulting fragment was cloned into *Sal*I-*Kpn*I digested pRY112 (Yao et al., 1993) and the complementation plasmid was introduced into the Δ*tlp2* deletion mutant by biparental conjugation as described (Miller et al., 2000). Transconjugants were selected on MH agar supplemented with kanamycin and chloramphenicol and the resulting complementation strain was designated *tlp2* comp as listed in Table 1.

### Chemotaxis Assay

To quantify chemotaxis, we adapted a modified capillary chemotaxis assay that quantitatively measures bacterial tactic responses (Mazumder et al., 1999; Cerda et al., 2003). The assay was previously used for quantifying chemotaxis in subsurface microaerophilic bacteria including *Campylobacter* (Mazumder et al., 1999; Chandrashekhar et al., 2015) and other *Epsilonproteobacteria,* such as *H. pylori* (Cerda et al., 2003, 2011). Briefly, *C. jejuni* wild type (WT), Δ*tlp2* mutant and the complemented strains were grown microaerobically at 42°C for 18 h on MH agar and resuspended in chemotaxis buffer (Phosphate Buffered Saline, PBS or Normal Saline, pH 7.4) and OD_600_ was adjusted to 0.5. A 100 μl volume of a solution of the compounds [All compounds at 0.1M except Pi (Inorganic Ventures, Christiansburg, VA, United States) at 1 mM and FeSO_4_ (Sigma) at 0.1 mM] to be tested for chemotaxis response (buffer alone served as control) was aspirated through a 22 G stainless-steel needle (0.254 mm diameter × 20 mm long) into a 1 ml tuberculin syringe. The 0.1 M concentration of the compounds was selected based on previous studies and a series of preliminary experiments that showed that 100 mM resulted in the strongest chemotaxis response (Vegge et al., 2009; Tareen et al., 2010). A 100 μl of the OD_600_ adjusted bacterial suspension was drawn into a 200 μl disposable pipette tip and the needle-syringe system was fitted to the pipette tip in such a way that the needle was immersed into the bacterial suspension. The system was positioned horizontally and incubated at 42°C for 1 h. The needle-syringe system was then separated from the bacterial suspension containing pipette tip and contents of the syringe were 10-fold serially diluted in chemotaxis buffer, plated onto MH agar plates and incubated at 42^o^C under microaerophilic conditions to determine colony-forming units (CFUs). Relative Chemotaxis Ratio (RCR) toward a test compound was ascertained as a ratio between the numbers of bacteria entering the test needle-syringes to those in the control needle-syringes. A test compound was considered as an attractant if the RCR was ≥ 2 (Mazumder et al., 1999). Results were expressed as the mean of three independent assays. A mutant was considered deficient in chemotaxis toward a substrate if both the corresponding RCR value was significantly < 2 (*P* < 0.05) and the CFU of the mutants were significantly lower (*P* < 0.05) than those of the wildtype. A *C. jejuni* 81–176 *cheY* mutant which is incapable of directional movement (negative control) (Yao et al., 1997) and 0.1% porcine gastric mucin (positive control; Sigma) were also used to evaluate the integrity of the assay. To test the response to repellents, *C. jejuni* cultures were mixed with a repellent and the bacteria that entered the syringe, which in this instance contained only buffer, to escape the repellent were quantified as described above. To account for any methodological bias, capillary chemotaxis results were further verified by using the disk method (Vegge et al., 2009) for selected compounds.

### Determination of the *tlp2* Transcriptional Levels With Reporter Gene Assays

The partial coding region of *tlp2* and the upstream region was amplified with *tlp2-*PF_F (*Sma*I) and *tlp2*-PF_R (*Bgl*II) primers and cloned into pMW10, a shuttle vector for *E. coli* and *C. jejuni,* containing a promoterless *lacZ* gene (Wosten et al., 1998). The plasmid was mobilized into *C. jejuni* WT and Δ*tlp2::Cm* strains by electroporation. The Δ*tlp2::Cm* strain was used for reporter studies since pMW10 carries kanamycin resistance. *The*Δ*tlp2::Cm* was generated as described above. β-Galactosidase assay was performed with *C. jejuni* strains harboring the *tlp2* promoter (P*_tlp2_*)-*lacZ* transcriptional fusion construct, as described previously (Wosten et al., 1998). To examine the effect of Pi and iron on *tlp2* transcription, reporter strains were incubated in MOPS and MEMα (Life technologies, Invitrogen) supplemented with Pi and FeSO_4_ or FeCl_3_ (Iron), respectively.

Additionally, reporter fusions were also created for the intergenic region between *tlp2* (*Cjj81176_0180*) and *phoX* (*Cjj81176_181*) to determine any potential promoter in the intergenic region. The intergenic region was amplified with specific primers listed in Table 2 and cloned into pMW10 using the *BamH*I*-Xba*I sites. Reporter gene assays were carried out as described above.

Reporter gene assays were also carried out in the *C. jejuni* 81–176 Δ*fur* mutant. *C. jejuni* 81–176 Δ*fur* mutant was created by natural transformation of WT containing the (P*_tlp2_*)-*lacZ* transcriptional fusion construct with genomic DNA from *C. jejuni* NCTC11168 Δ*fur* mutant as described previously (Jeon et al., 2008; Gangaiah et al., 2009). Briefly, 1 ml of *C. jejuni* WT reporter strain was resuspended to an OD_600_ of 0.5. Approximately, 5 μg of genomic DNA from *C. jejuni* NCTC11168 Δ*fur* mutant was added and incubated for 4 h microaerobically. The bacteria were plated on MH plates supplemented with appropriate antibiotics and incubated microaerobically at 42^o^C for 48 h. The deletion of the *fur* gene in 81–176 was confirmed by PCR.

### RNA Extraction and Reverse Transcriptase Overlapping PCR

Briefly, *C. jejuni* WT grown overnight in MH agar plate was scraped and resuspended to an OD_600_ of 0.05 in MEM-α or MH broth and grown up to mid log phase (6 h), respectively. Total RNA was extracted using RNeasy Mini Kit (Qiagen) and quantified using NanoDrop ND-2000c spectrophotometer (Wilmington, DE, United States). cDNA synthesized using SuperScript^®^ III First-Strand Synthesis SuperMix (Invitrogen), was used as a template for PCR with a set of overlapping primers for the *tlp2, phoX,* and the 135 base pairs intergenic region between *tlp2* and *phoX*. (Table 2).

### Primer Extension Assay

Primer extension assay was performed as described previously (Kim et al., 2011). Briefly, *C. jejuni* WT strain was grown for 6 h (mid-log phase) with shaking in MH broth at 42^o^C and harvested by centrifugation at 10,000 × *g* for 5 min. Total RNA was purified with TRIzol (Invitrogen) according to the manufacturer’s instructions. Purified RNA was resuspended in sterile distilled RNase-free water, and the RNA concentration was determined by measuring the OD of the solution at 260 and 280 nm using NanoVue (GE Healthcare). A portion (10 pmol) of the PE_R primer was labeled with ^32^P at the 5′ end by 10 U of T4 polynucleotide kinase (Invitrogen) and 80 μCi of [γ-^32^P] dATP for 30 min at 37°C. The labeling mixture was heated at 70°C for 10 min and purified with MicroSpin G-25 columns (GE Healthcare). The γ-^32^P-end-labeled primer (0.5 pmol) was co-precipitated with 15 μg of total RNA by the addition of sodium acetate and absolute ethanol. The pellet was washed with 75% ethanol, dried at room temperature, and resuspended in 20 μl of 250 mM KCl, 2 mM Tris (pH 7.9), and 0.2 mM EDTA. The mixture was heated to 65°C and then was allowed to cool to room temperature for 1 h. After annealing, 50 μl of reaction solution containing 5 μg of actinomycin D, 700 μM deoxynucleoside triphosphates, 10 mM MgCl_2_, 5 mM DTT, 20 mM Tris (pH 7.6), 30 U of RNasin (Promega), and 150 U of Superscript^®^ III reverse transcriptase (Invitrogen) was added. The mixture was incubated at 42°C for 70 min and treated with 100 U of RNase T1 (Invitrogen) at 37°C for 15 min. The sample was ethanol precipitated after addition of 1.4 μl of 5M NaCl with 2.5 volumes of absolute ethanol and then washed with 75% ethanol. Sample was resuspended with 6 μl of formamide dye and 4 μl of Tris-EDTA (pH 8.0) buffer and then denatured at 90°C for 3 min. The samples were resolved on 6% polyacrylamide-8M urea gels, and the reverse transcription signals were analyzed by using BAS 2500 (Fuji Film). Primers, CJJ81176_180_PE and CJJ81176_181_PE (Table 2) were used for sequencing the upstream regions of *tlp2* and *phoX,* for transcription start site with a SequiTherm EXCELII DNA sequencing system (Epicenter).

### Alkaline Phosphatase Assay

PhoX activity was determined as described previously (Drozd et al., 2011). Briefly, WT, Δ*tlp2,* and the *tlp2 comp* strains were grown overnight on MH plates with appropriate antibiotics. The cultures were gently scraped, washed and resuspended in MEM and incubated at 42°C microaerobically with shaking for 2 h. Cultures were then centrifuged for 10 min at 7000 × *g* and supernatant was removed. Cells were gently washed with 50 mM MOPS buffer (pH 7.4) (Sigma) and incubated with shaking at 42^o^C for 2 h following which, OD_600_ readings were taken. Cells were pelleted and resuspended in PNPP buffer containing 2 mM p-nitrophenyl phosphate (PNPP; Sigma) and incubated at 37°C. OD measurements at 550 nm and 420 nm were taken, and the phosphatase activity was calculated as described previously (Wosten et al., 2006). The assay was performed a total of three times with duplicate samples in each assay. Additionally, effect of iron on PhoX activity was assessed by supplementation of FeSO_4_ at 40 μM concentration in MOPS buffer.

### Nutrient Downshift Assay

The role of *tlp2* in *C. jejuni* survival under nutrient downshift was assessed using MEM-α as described previously (Gangaiah et al., 2010). Briefly, mid-log-phase cultures of WT, Δ*tlp2,* and the *tlp2 comp* strains were pelleted, washed twice and resuspended in MEM-α with OD_600_ adjusted to 0.05. The bacterial suspensions were incubated microaerobically at 42°C with shaking. Samples were taken over time, serially diluted (10-fold) in MEM-α media and plated on MH agar for determining CFU. The experiment was performed three times and the average for each time point was taken.

### Quantitative Reverse Transcriptase PCR (qRT-PCR) Analysis of Phosphate Uptake Genes

The *C. jejuni* WT and Δ*tlp2* cultures were assessed for changes in expression of phosphate uptake genes (*phosR, pstC,* and *pstS*) (Wosten et al., 2006). Briefly, *C. jejuni* WT and Δ*tlp2* strains were grown to mid-log phase in MEM-α microaerobically, with shaking at 42^o^C. Total RNA was extracted using RNeasy Mini Kit (Qiagen) and cDNA was synthesized using SuperScript^®^ III First-Strand Synthesis SuperMix (Invitrogen). RNA and cDNA concentrations and purity were determined using NanoDrop ND-2000c spectrophotometer (Wilmington, DE, United States). Quantitative RT-PCR was performed with a SensiMixPlus SYBR RT-PCR kit (Quantace, Norwood, MA, United States) in a Mastercycler ep realplex2 thermal cycler (Eppendorf, Westbury, NY, United States). Gene specific primers (Table 2) used in this analysis have been described previously (Drozd et al., 2014). The relative levels of expression of target genes were normalized to 16S rRNA gene expression of the same strain. The relative fold changes in gene expression was calculated using the comparative threshold cycle (CT) method to yield fold-difference in transcript level compared to WT (Livak and Schmittgen, 2001). The qRT-PCR was performed a total of three times with duplicate samples in each assay.

### Invasion and Intracellular Survival Assays

Invasion and intracellular survival of *C. jejuni* WT and Δ*tlp2* mutant in INT 407 cell line (human embryonic intestine cells, ATCC CCL 6) was assessed as described previously (Kassem et al., 2012). Briefly, mid-log phase grown bacterial cells were collected by centrifugation (5,000 × *g,* 10 min), washed twice with MEM containing 1% (v/v) FBS and resuspended in MEM. INT 407 cells (1.4 × 10^5^ per well) in MEM with 10% (v/v) fetal bovine serum (FBS) were seeded in 24-well tissue culture plate and incubated for 18 h at 37°C with 5% CO_2_. INT 407 cells were infected with multiplicity of infection (MOI) 100 for invasion and intracellular survival assays and incubated for 3 h at 37°C. Following 3 h of incubation with bacteria; cells were treated with gentamicin (150 μg/ml) and incubated for additional 2 h. After 2 h of incubation, the infected cells were rinsed three times with MEM, lysed with 0.1% (v/v) Triton-X 100, serially diluted in MEM and plated on MH agar. The percent invasion was calculated as follows: (no. of CFU recovered after lysis of INT 407 cells/CFU added to each well) × 100.

To assess survival of *C. jejuni* WT and Δ*tlp2* mutant in INT 407 cell line, following 2 h of gentamicin treatment, the infected cells were washed with MEM three times and covered with MEM containing gentamicin (10 μg/ml) and incubated for 24 h at 37°C. After 24 h of incubation, infected cells were washed with MEM, lysed and plated as described above. In parallel, we also cultured the supernatant of gentamicin treated monolayers to ensure the quality of the gentamicin protection assay.

### Chicken Colonization Assay

Chicken colonization study was performed as described previously (Gangaiah et al., 2009). Briefly, 3 day-old specific pathogen free chickens (*n* = 6 for each group) were obtained from a local hatching facility (Food Animal Health Research Program, OARDC, Wooster, OH, United States). *Campylobacter* free chickens were inoculated orally with 10^4^ CFU of the *C. jejuni* WT and Δ*tlp2* mutant strain in 200 μl of PBS (pH 7.4). Chickens were euthanized after 7 days post-inoculation and ceca, duodenum, jejunum, liver, spleen and bursa were collected aseptically, weighed, homogenized, serially diluted in PBS (pH 7.4) and plated on appropriate MH agar containing *Campylobacter* selective supplement with or without kanamycin to determine colony forming units (CFU). Plates were incubated at 42°C microaerobically and CFUs per gram of tissues were determined.

### Statistical Analysis

Statistical significance of data generated in this study was determined using two tailed Student’s *t*-test. Results of the promoter fusion assay were statistically analyzed using one way Anova with Dunett’s multiple comparison posttests. Data from the chicken colonization experiment was analyzed using the Mann Whitney test. *P* ≤ 0.01 or 0.05 (α level) was considered statistically significant.

## Results

### The Δ*tlp2* Mutant Is Defective in Chemotaxis Toward Aspartate, Pyruvate, Pi and Iron

**FIGURE 1:**
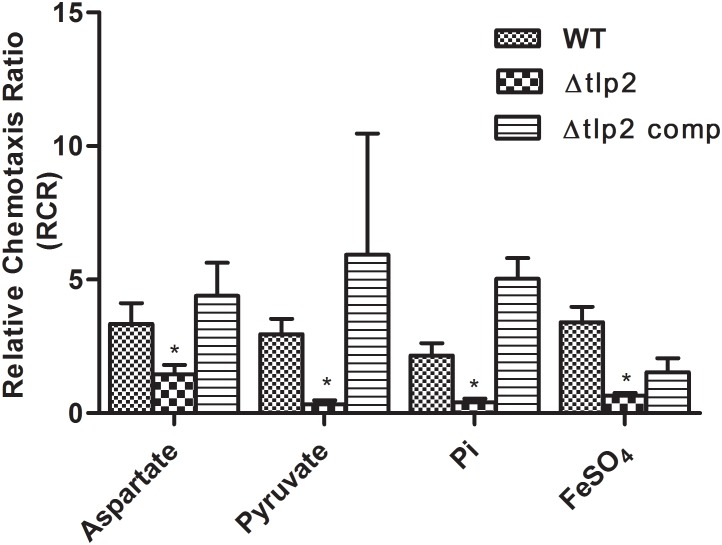
The Δ*tlp2* mutant was significantly defective in chemotaxis toward aspartate, pyruvate, inorganic phosphate and ferrous sulfate. Chemotaxis was determined using the capillary method (Chandrashekhar et al., 2015) where an RCR-value of 2 or above indicates chemotaxis toward the test chemical. This graph represents only those compounds (out of 15 compounds) for which a defect in chemotaxis was observed in the Δ*tlp2* mutant. The complete results for chemotaxis toward all 15 compounds tested are listed in Table 3. ^∗^*P* ≤ 0.05. The results show the means and standard errors of three independent experiments.

To assess the role of *tlp2* in *C. jejuni* chemotaxis, a deletion mutant was constructed with the coding region of *tlp2* being replaced with kanamycin resistance gene. Syringe capillary chemotaxis assays were performed to determine the chemotactic activity of *C. jejuni* WT, the Δ*tlp2* mutant and the complemented strains toward different substrates (Table 3). Substrates with RCR values > 2 and < 0.1 were considered as chemo attractants and repellants, respectively, for WT *C. jejuni* (Hugdahl et al., 1988; Cerda et al., 2003; Chandrashekhar et al., 2015). In addition, capillary assay showed strong chemotaxis of *C. jejuni* toward 0.1% porcine gastric mucin (RCR = 9.0), while a non-motile *cheY* mutant had an RCR below the detection limit (∼0) for some of the known attractants (Chandrashekhar et al., 2015). Compared to the WT, the Δ*tlp2* mutant was defective in chemotaxis toward aspartate (*P* = 0.0292) and pyruvate (*P* = 0.0010) with RCR values < 2 (Figure 1) (RCR values: aspartate: 3.81 for the WT and 1.45 for the Δ*tlp2* mutant; pyruvate: 2.96 for the WT and 0.33 for the Δ*tlp2* mutant) (Cerda et al., 2003). Even though the *tlp2* mutant showed RCR values less than 2 for isocitrate, succinate and propionate; they were not statistically significant (Table 3). Interestingly, Δ*tlp2* mutant also showed a chemotaxis defect toward Pi and iron (FeSO_4_), compared to WT (Figure 1). We observed that FeSO_4,_ but FeCl_3_.6H_2_0 (ferric iron source) and (NH_4_)_2_SO_4_ (sulfate source) were not a chemoattractant for *C. jejuni,* based on the RCR indices for these compounds (FeCl_3_.6H_2_0: RCR of 1.40 and (NH_4_)_2_SO_4_: RCR of 0.40) (Table 3). The chemotaxis defect was restored to WT levels in the complemented strain; however, chemotaxis toward iron was partially restored in the complemented strain (Figure 1). Chemotaxis results were also confirmed by assessing chemotaxis using the disk method for selected substrates such as aspartate and pyruvate (data not shown).

**Table 3:** RCR values^a^ for the WT and *tlp2* mutant for all compounds tested.

Chemicals tested	WT	Δ*tlp2*
Aspartate	3.81 ± 0.32	1.45 ± 0.35
L-glutamine	4.19 ± 0.77	2.93 ± 0.38
L-serine	2.04 ± 0.46	1.99 ± 0.82
Fumarate	9.32 ± 1.45	2.52 ± 0.79
Isocitrate	2.51 ± 0.41	1.5 ± 0.65^b^
Formate	4.41 ± 0.13	2.36 ± 1.05
Succinate	3.73 ± 1.2	1.2 ± 0.70^b^
Pyruvate	2.96 ± 0.566	0.33 ± 0.15
Propionate	2.97 ± 1.51	1.35 ± 0.30^b^
Inorganic phosphate	2.15 ± 0.46	0.4 ± 0.15
Deoxycholic acid	<0.1	<0.1
Cholic acid	<0.1	<0.1
FeSO_4_	3.40 ± 0.58	0.66 ± 0.10
FeCl_3_.6H_2_0^a^	1.4 ± 0.35	NT
(NH_4_)_2_SO_4_^a^	0.1	NT

*The results show the means and standard errors of three independent experiments. An RCR value of 2 or above indicates chemotaxis toward the test chemical (Mazumder et al., 1999). ^a^The RCR value of the WT strain was <2.0, hence the compounds were not chemoattractants for C. jejuni 81–176 and were therefore not tested (NT) for chemotaxis with the mutant. ^b^The RCR of these compounds are lesser than 2 for the tlp2 mutant, however, not significant statistically P > 0.05.*

### Iron Induces *tlp2* Promoter (P*_tlp2_*) Activity

**FIGURE 2:**
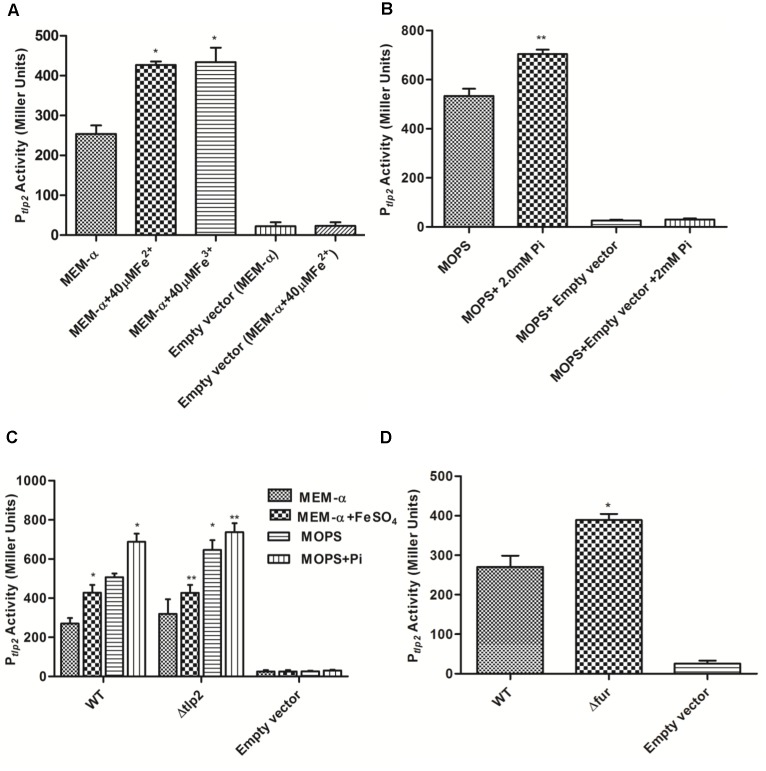
β-galactosidase activity of *Campylobacter jejuni* WT carrying P*_tlp2_*-*lacZ* transcriptional fusion construct. **(A)** β-galactosidase activity in the absence (uninduced) and presence of 40 μM FeSO_4_ or FeCl_3_(H_2_O)_6_ (induced) added to MEM-α. **(B)** β-galactosidase activity in the absence (uninduced) and presence of 2mM Pi (induced) added to MOPS. **(C)** β-galactosidase activity of the P_tlp2_-lacZ fusion assays in the Δ*tlp2::Cm* mutant in the presence or absence of 40 μM FeSO_4_ in MEM-α and in the presence or absence of 2mM Pi in MOPS. **(D)** β-galactosidase activity of the P_tlp2_-lacZ fusion in the Δ*fur* mutant in MEM-α. The cells were incubated for 8 h before carrying out the assay. The results show the means and standard deviations of three independent experiments. ^∗^*P* < 0.05 where each group is compared with the WT reporter strain that is not induced (MEM-α or MOPS) and *^∗∗^P* < 0.05 where each group is compared with the WT that is induced (with FeSO_4_ or Pi).

Decreased chemotaxis toward iron observed in the Δ*tlp2* mutant encouraged us to investigate the *tlp2* expression under different growth conditions. The level of *tlp2* transcription was quantified with β-galactosidase assays in presence of metal ions, such as Fe^2+^, Fe^3+^, Cu^2+^, Ca^2+^, Mg^2+^ and Zn^2+^ in MEM-α that does not contain these metals (van Vliet et al., 1998; Kim et al., 2011). Assay was performed in the presence of 20 μM CuCl_2_, 40 μM FeSO_4_, 40 μM FeCl_3_, 40 μM MnCl_2_, and 10 μM ZnCl_2._ Since MEM-α media already has Ca^2+^ (1.8 mM) and Mg^2+^ (0.8 mM), we did not supplement the media with these two metal ions. Iron in both ferrous (FeSO_4_) and ferric (FeCl_3_) forms induced *tlp2* expression at 40 μM concentrations (Figure 2A), whereas other metals had no effect on the level of *tlp2* transcription (Supplementary Figure S1A). For Fe^2+^, 40 μM was used based on the dose response assay (Supplementary Figure S1B) which showed best result at this concentration (Supplementary Figure S1B). Concentrations of iron as low as 5 μM FeSO_4_ also significantly induced *tlp2* expression (Supplementary Figure S1B).

Similarly, the activity of P*_tlp2_* was investigated in the presence of Pi due to the observed chemotaxis defect toward Pi (Table 3 and Figure 1). MOPS buffer was used as a low phosphate medium for the incubation of the *C. jejuni* reporter strains (Gangaiah et al., 2009). The concentration of Pi added to MOPS buffer ranged from 1 to 3 mM but P*_tlp2_* was most significantly induced in the presence of 2 and 3 mM of Pi (Figure 2B and Supplementary Figure S1C). Further, P*_tlp2_* activity in the *tlp2* deletion mutant was also studied to assess the effect of the gene product on its promoter activity. Since pMW10 shuttle vector has a kanamycin resistant cassette, we created a Δ*tlp2* mutant with a chloramphenicol resistant cassette. We found that the *tlp2* expression was also induced in the Δ*tlp2* mutant in the presence of Pi and FeSO_4_ similar to WT (Figure 2C). Even though the P*_tlp2_* activity in Δ*tlp2* mutant was higher than the WT both in the presence or absence of Pi and Fe, the difference was not statistically significant. These results suggest that *tlp2* transcription is independent of Tlp2 protein levels in the cell.

Ferric uptake regulator protein (Fur) plays an important role in *C. jejuni* iron homeostasis (van Vliet et al., 1998). In addition, iron and Fur are shown to regulate *tlp* genes (Cj0262c; Tlp4 and Cj1110c; Tlp8) in *C. jejuni* (Butcher et al., 2012). We, therefore, investigated if *tlp2* expression is regulated by Fur. Interestingly, the P*_tlp2_* activity was increased in a Δ*fur* mutant of *C. jejuni* 81–176 in MEM-α (Figure 2D). These observations revealed a role for *fur* in the regulation of *tlp2* expression. Since a *fur* mutation derepresses genes involved in iron acquisition in *C. jejuni* (Holmes et al., 2005), there will be over-accumulation of iron in the *fur* mutant. The increased levels of intracellular levels may increase the P*_tlp2_* activity in the *fur* mutant.

### The *tlp* and *phoX* Genes Are Co-transcribed

**FIGURE 3:**
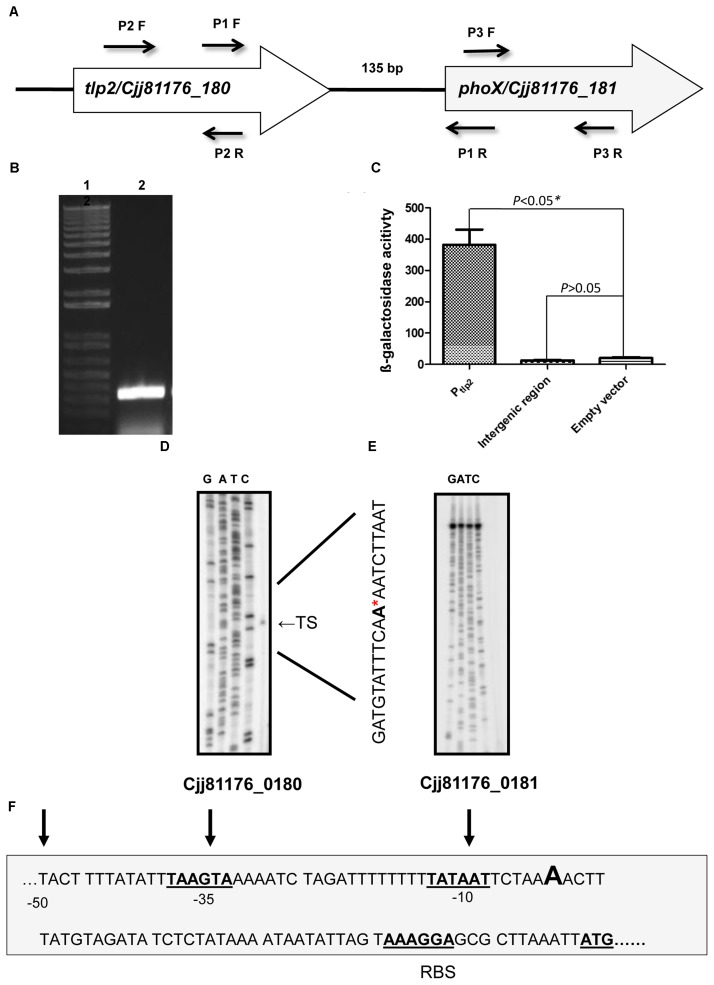
**(A)** Genetic organization of *tlp2.* The *tlp2* gene (*cjj81176_180*) is located upstream of the *phoX* gene (*Cjj81176_181*) which encodes the alkaline phosphatase (PhoX) enzyme. The *tlp2* and *phoX* genes are separated by a 135 bp intergenic region. **(B)** Reverse Transcriptase overlapping PCR showing co-transcription of *tlp2* and *phoX.* Intergenic region was amplified with primer pair P1 F (forward) and R (reverse) in WT strain grown in MEM-α. Primers for the *tlp2* (P2 F and R) and *phoX* (P3 F and R) genes were included as control regions as well (data not shown). **(C)** The P*_int_-lacZ* fusion showed no β-galactosidase activity compared to the P*_tlp2_*–*lacZ* fusion in the WT strain. Determination of the transcriptional start site for *tlp2* **(D)** and *phoX* **(E)** by a primer extension assay. Only one transcriptional start site is seen upstream to *tlp2,* designated TS (Transcriptional Start) indicated with an arrowhead on the right and by the ^∗^ in the sequence. No transcriptional start site was found in the region upstream to *phoX* **(F)**: The −10 and −35 elements of the P*_tlp2_* are underlined and the ribosomal binding site is indicated as RBS.

The *tlp2* gene (CJJ81176_180) is located upstream to *phoX* (CJJ81176_181) in the same orientation with an intergenic region of 135 base pairs (Figure 3A). Additionally, a previous study indicated that *phoX* gene (Cj0145), located immediately downstream of *tlp2,* is induced by iron and was also enriched in the CjFur ChIP-chip assay (Butcher et al., 2012). As the Δ*tlp2* mutant is defective in chemotaxis toward iron (Fe) and Pi, and the *tlp2* transcription is modulated by iron and Pi, we hypothesized that these two genes may be co-transcribed. To test this, total RNA was extracted from WT grown in MEM-α and analyzed by RT-PCR using primers designed to amplify a flanking region of the two genes. The results indicated that *tlp2* and *phoX* are co-transcribed (Figure 3B). In addition, an amplicon was also observed when WT was grown in nutrient rich MH broth (data not shown). These results implied that *phoX* is co-transcribed with *tlp2* under the conditions tested in this study.

Further, an intergenic region between *tlp2* and *phoX* was fused to the promoterless *lacZ* gene to confirm that the promoter activity observed was specific to P*_tlp2_*. The reporter strains did not show any promoter activity; the promoter activity in the β-galactosidase assay was similar to that of the negative control (empty plasmid) (Figure 3C). Similar findings were observed when media were supplemented with iron or Pi (data not shown), confirming that there was no promoter in the intergenic region between *tlp2* and *phoX* under these tested conditions.

Furthermore, a primer extension analysis revealed a single transcription start site (TS) upstream to the *tlp2* gene (Figures 3D,F). The TS is located 53 bp upstream of the *tlp2* start codon with a ribosomal binding site located 12 bp upstream from the start codon. The −10 region was identified with the first T of the TATA box located 59 bp upstream of the start codon. Consistent with the results above, no transcription start site was observed in the 135 bp intergenic region between *tlp2* and *phoX* (Figure 3E). This result indicates that *tlp2* and *phoX* genes constitute an operon, and the transcription of *phoX* is dependent on the *tlp2* promoter and they are co-transcribed.

### Alkaline Phosphatase Activity Is Increased in the Presence of Iron

**FIGURE 4:**
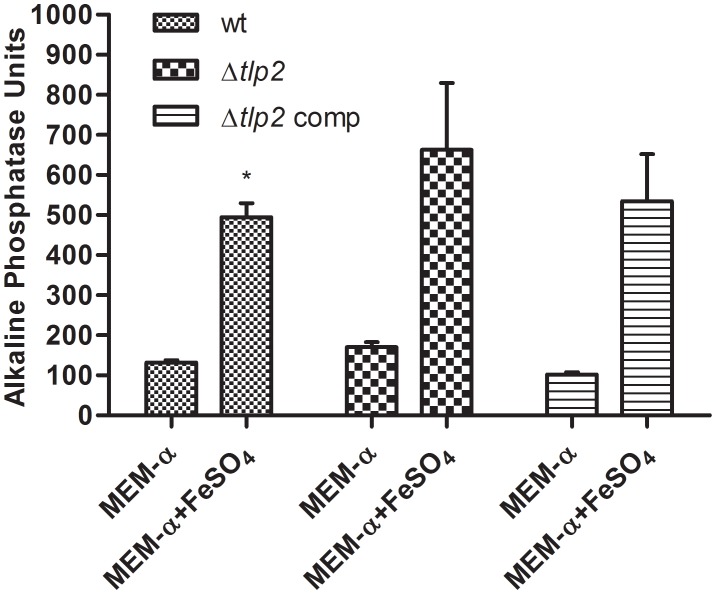
*C. jejuni* WT and Δ*tlp2* strains shows a higher PhoX activity in the presence of iron (40 μM FeSO_4_ ). The bacterial cultures grown in MEM-α in the presence or absence of 40 μM FeSO_4._ The data is a mean of 3 independent trials. ^∗^*P* ≤ 0.05.

A study investigating the regulatory potential of Fur of *C. jejuni* identified that *phoX* is activated by iron (Butcher et al., 2012). Therefore, the PhoX activity of *C. jejuni* WT was evaluated in MEM-α supplemented with 40 μM FeSO_4_. The PhoX activity of the WT strain increased approximately four-fold in the presence of iron (Figure 4). Similarly, a higher PhoX activity was also observed in the Δ*tlp2* mutant in MEM-α in the presence of iron similar to the WT (Figure 4); however, this increase was not significant (*P* ≤ 0.09). In the complemented strain, the PhoX activity was similar to the WT with or without iron (Figure 4). These results suggest that iron upregulates the PhoX activity in *C. jejuni* and potentially intersects the phosphate utilization pathway of *C. jejuni*.

### Deletion of *tlp2* Affected Nutrient Stress Survival

**FIGURE 5:**
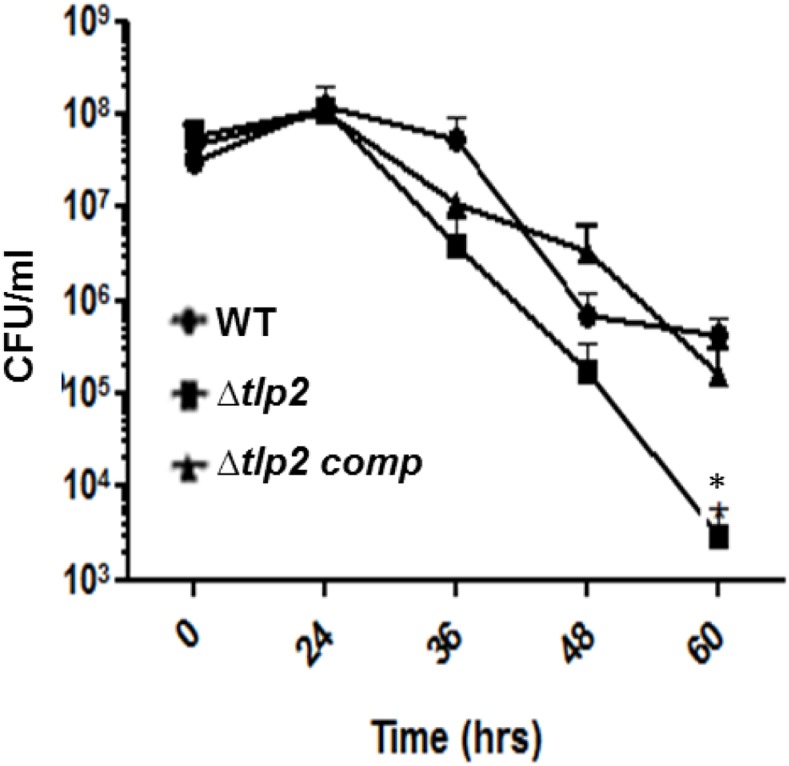
Survival of Δ*tlp2* mutant under nutrient downshift. Survival under nutrient stress was assessed by resuspending MH agar grown bacterial cultures in MEM-α, and determining CFU at different time points. Each data point represents the mean ± SE of 3 independent experiments. ^∗^*P* ≤ 0.05.

The effect of a *tlp2* mutation on stress survival was monitored by comparing the growth of the Δ*tlp2* mutant strain to the WT *C. jejuni* in nutrient-limited conditions. The Δ*tlp2* mutant did not display any growth defect when grown in nutrient-rich MH broth (data not shown); however, the *tlp2* mutant on transition from nutrient rich MH broth to nutrient deficient MEM (without glutamine) exhibited survival defects in the late stationary phase especially 36 h and onward. The survivability of the *tlp2* mutant strain was decreased by one and more than two orders of magnitude at 36 and 60 h, respectively, as compared to the WT (*P < 0.*05) (Figure 5).

### Deletion of *tlp2* Affected Intracellular Survival in Intestinal Epithelial Cells

**FIGURE 6:**
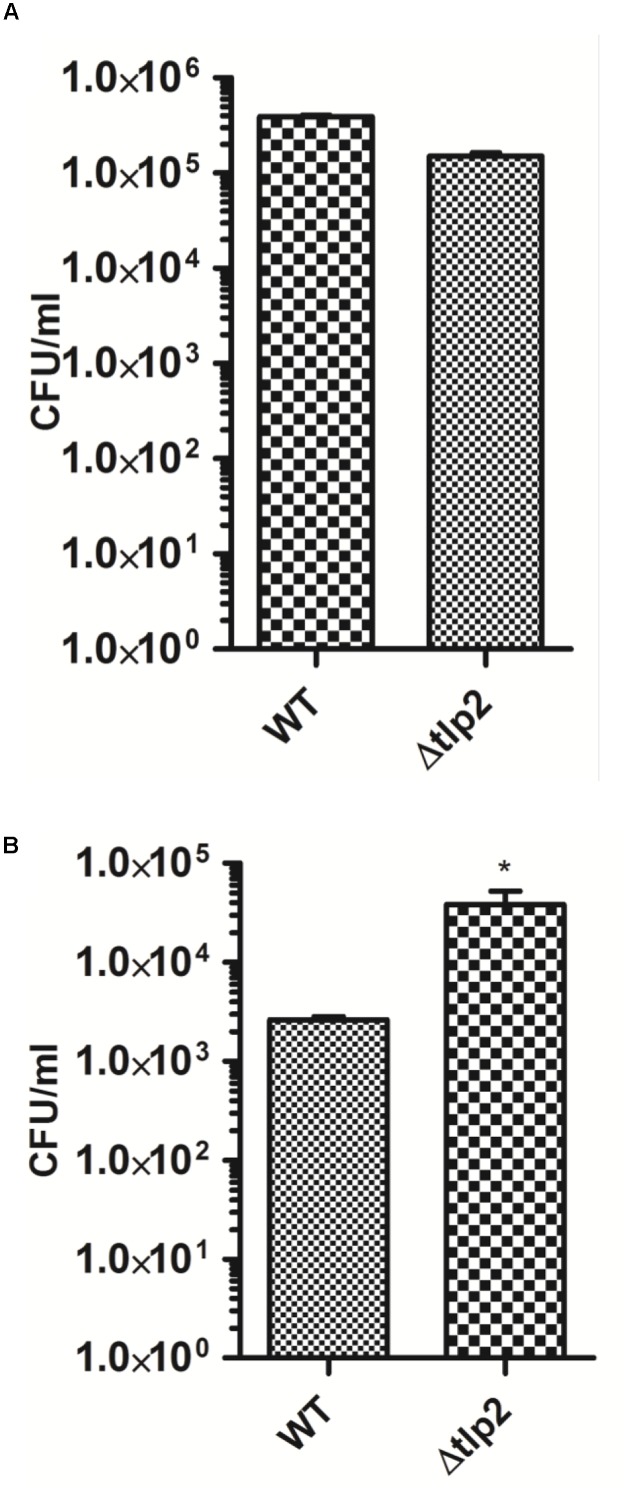
The Δ*tlp2* mutant exhibits no invasion defect **(A)** and increased intracellular survival **(B)** in INT 407 human intestinal cells. Bacteria were infected at an MOI of 100 for both invasion (2 h of gentamicin treatment) and intracellular survival assays (24 h of further incubation). Each data point represents the mean ± SE of 3 independent experiments. ^∗^*P* ≤ 0.05.

The consequence of *tlp2* deletion on virulence-associated traits of *C. jejuni* was evaluated by the ability of Δ*tlp2* mutant to invade and survive within the human intestinal epithelial INT 407 cells (Candon et al., 2007). The Δ*tlp2* demonstrated similar invasion in INT 407 cells; however, the Δ*tlp2* mutant showed a higher intracellular survival, with almost 2 logs more bacteria recovered compared to the WT (Figures 6A,B).

### The Δ*tlp2* Mutant Is Defective in Colonization of the Chicken Gastrointestinal Tract

**FIGURE 7:**
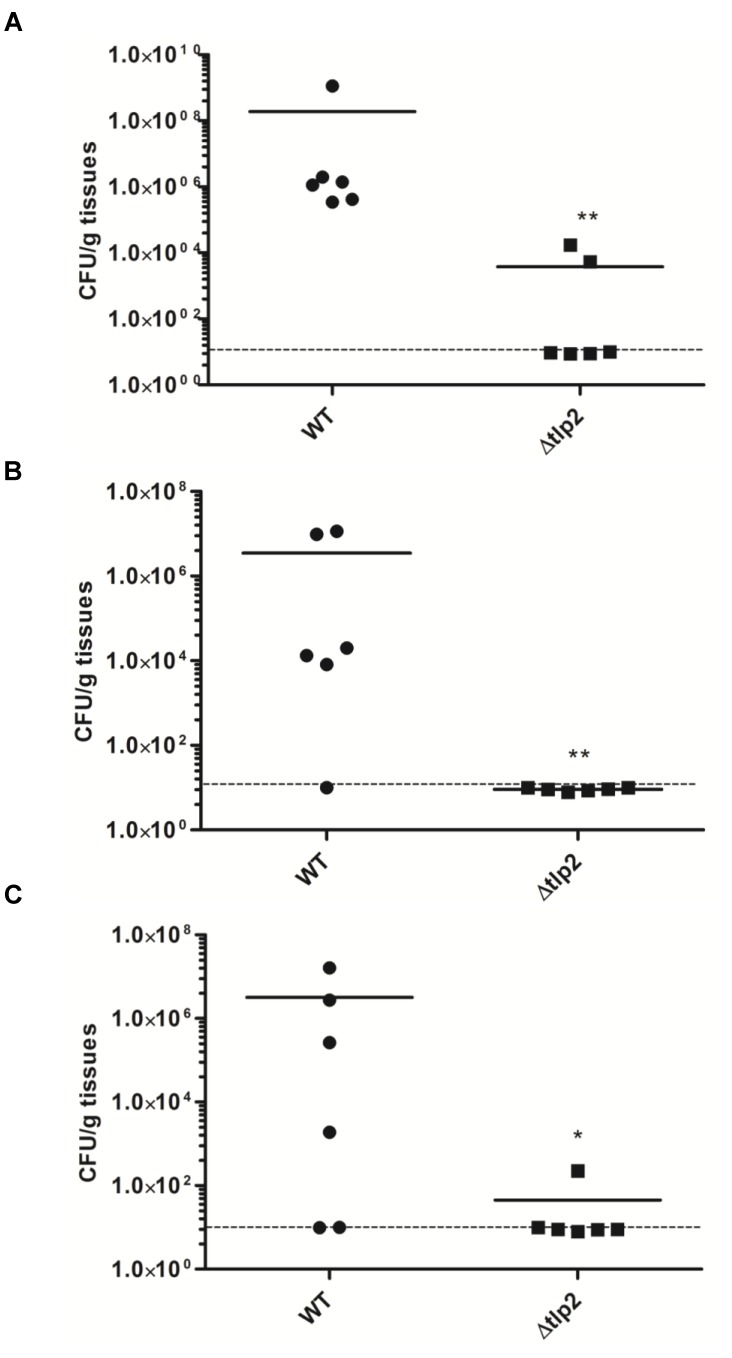
Colonization of the Δ*tlp2* mutant in chicken gastrointestinal tract. The Δ*tlp2* mutant exhibited a significant defect in colonization of the **(A)** cecum **(B)** duodenum and **(C)** jejunum, compared to the WT. Each data point represents CFU/g of tissue. ^∗^*P* ≤ 0.05 and ^∗∗^*P* ≤ 0.01 Dotted line represents the detection limit.

To investigate the role of Tlp2 in colonization of *C. jejuni,* we investigated the colonization of Δ*tlp2* mutant and WT in different segments of the chicken intestine. The Δ*tlp2* mutant and WT were inoculated into 3-day old chicks orally (10^4^ CFU/chicken), and bacterial burden was analyzed after 7 days of infection. Colonization of the *C. jejuni* WT strain in the chicken gastrointestinal tract (cecum and duodenum/jejunum) ranged from 4 × 10^7^ to 2 × 10^8^ CFU per gram of tissue; while in the Δ*tlp2* mutant varied from 2 × 10^2^ to 8 × 10^3^ CFU per gram of tissue. The Δ*tlp2* mutant showed a 4–5 logs decrease in cecal colonization compared to the WT (Figure 7A). The Δ*tlp2* mutant was not detected in the duodenum and colonization of the jejunum was also reduced by almost 4 logs (Figures 7B,C). However, the liver, spleen, and bursa showed no colonization by *C. jejuni* WT and Δ*tlp2* mutant. These findings suggest that the *tlp2* is essential for achieving optimal colonization in the proximal and distal segments of the gastrointestinal tract, including the cecum.

## Discussion

In this study, we characterized the role of *tlp2* in chemotaxis, stress survival, and colonization of the chicken gut. Our results indicated that *tlp2* is involved in chemotaxis toward aspartate, pyruvate, Pi, and iron. Promoter fusion assays revealed that iron, in the ferrous and ferric form induces the *tlp2* promoter activity. Iron is essential for *C. jejuni* colonization in the host as it is one of the limiting nutrients sequestered away from the pathogen by the host and the bioavailability of iron in the intestine is not very well understood (Naikare et al., 2006).

Predicted domain structure of the Tlp2 in the SMART database (Schultz et al., 1998) revealed a single periplasmic Cache_1 (Ca^2+^ channels and chemotaxis receptors) domain (Anantharaman and Aravind, 2000) and a cytoplasmic MCP signaling domain. Cache domain is found in the extracellular or periplasmic portions of chemoreceptors from Gram-positive and Gram-negative bacteria, and is associated with sensing of small molecules (Anantharaman and Aravind, 2000). The cache domains of *Pseudomonas aeruginosa* and *Vibrio cholerae* have been associated with chemotaxis toward amino acids (Nishiyama et al., 2012). The Cache domain is responsible for interaction with multiple ligands and thereby chemotaxis (Tasneem et al., 2005). Δ*tlp2* mutant shows decreased chemotaxis toward aspartate, pyruvate, iron and Pi. Additionally, Tlp2 shows 38% identity with the periplasmic region of the multiple ligand binding Tlp3 (Ccml) of *C. jejuni,* possessing a single cache domain which can potentially bind to multiple ligands with varying affinity (Rahman et al., 2014).

Studies in *S. oneidensis* and *G. metallireducens* report chemotaxis toward iron in the ferrous form (Childers et al., 2002; Bencharit and Ward, 2005). Iron is a redox active metal, and chemotaxis to iron suggests bacterial movement through reduced metal gradients toward potential electron acceptors (oxidized ferric form). Our observations in the WT (*C. jejuni* 81–176) strain show that it is chemotactic toward ferrous iron. The chemotactic response of *C. jejuni* toward iron (Fe^2+^) can be explained as bacterial adaptation to the assimilatory requirement for iron, as it is an important constituent of iron sulfur proteins and other cellular processes (Bencharit and Ward, 2005). Comparably, the chemotactic response of *H. pylori* toward a metal ion (zinc) has been primarily attributed to the mechanism of nutrient acquisition by bacteria (Sanders et al., 2013).

A study in *C. jejuni,* employing CjFur ChIP-chip analysis, identified *cj0145* (*phoX*) as a novel gene in the Fur regulon in *C. jejuni,* which is activated by iron (Butcher et al., 2012). Much in line with the study above, we found in our study that PhoX activity in the WT is significantly increased in the presence of iron. A recent study on the *Pseudomonas fluorescens* PhoX revealed that iron is a cofactor required for enzyme activity, additionally implying that the bioavailability of iron affects bacterial phosphate uptake (Yong et al., 2014). Although a similar mechanism for increased PhoX activity in *C. jejuni* in the presence of iron can be envisioned, a further biochemical investigation on *C. jejuni* PhoX is needed to identify the precise role of iron in its enzymatic activity.

The sensing, uptake and utilization of inorganic phosphate in prokaryotes enables their ability to withstand conditions of phosphate deprivation. The Pi sensing or taxis has been studied in bacterial pathogens such as *Enterococcus cloacae* and *P. aeruginosa* under conditions of phosphate starvation, with two chemotactic transducers identified for Pi taxis in *P. aeruginosa.* The Pho regulon and the phosphate uptake system regulate Pi taxis in both bacteria (Kusaka et al., 1997; Wu et al., 2000). *C. jejuni* being an enteric pathogen is subjected to its survival under low phosphate conditions in the chicken gastrointestinal tract. While the uptake and utilization of Pi in *C. jejuni* through the two-component PhosS/PhosR operon has been previously described (Wosten et al., 2006), nothing is known about Pi taxis in this microaerophile. In our study, *C. jejuni* WT is chemotactic toward Pi, whereas the Δ*tlp2* mutant displayed a decreased chemotaxis. The decreased cellular availability of Pi in the *tlp2* mutant was accompanied by an upregulation of the *phosR* (response regulator of Pho regulon) and the genes for phosphate uptake (*pstC* and *pstS*) which is normally induced in response to Pi limitation (Supplementary Figure S2) (Wosten et al., 2006). Additionally, the *tlp2* mutant’s decreased survival under nutrient mediated stress (Figure 5) can be attributed to the Pi limiting conditions created due to decreased Pi taxis. Earlier studies have indicated that survival under low-nutrient stress is regulated by PPK1 mediated synthesis of poly-P from Pi (Candon et al., 2007; Gangaiah et al., 2009).

PhoX hydrolyzes phospho-organic compounds to Pi, a preferred phosphate source and a building block for poly-P in *C. jejuni* (Candon et al., 2007; Drozd et al., 2011). PhoX in *C. jejuni* is activated by the PhosS-PhosR two component system, under phosphate limiting conditions (Wosten et al., 2006). However, what remains to be investigated is whether PhosR also regulates the *tlp2* promoter activity in *C. jejuni*. The *tlp2* gene is located upstream to *phoX* in *C. jejuni,* and our investigation of *tlp2* transcriptional organization revealed that both genes are transcribed together from a single promoter (P*_tlp2_*) located upstream to *tlp2*. These findings however, contradict a previous finding in *C. jejuni* 81116, where *phoX* was shown to be transcribed by a promoter located in the intergenic region of *tlp2* and *phoX,* when *C. jejuni* was grown in a chemically defined medium (Wosten et al., 2006). However, we could not observe any promoter activities in the intergenic region using a primer extension assay under our experimental conditions (Figure 3). Further, both strains possess 135 bps intergenic region between *tlp2* and *phoX*; however, showed 95.5% sequence similarity. Therefore, the disparity could be due to the different media and strains used in the two different studies.

The Δ*tlp2* mutant exhibited an increased intracellular survival in INT 407 cell monolayer than the WT strain. The group A Tlps 1, 4, 7 and 10 but not Tlp2, have been shown to play a role in *C. jejuni* invasion of human intestinal epithelial cells (Vegge et al., 2009; Hartley-Tassell et al., 2010; Tareen et al., 2010). *C. jejuni* is known to survive within epithelial cells and can be viable for up to 24 h (Watson and Galan, 2008). Studies have also indicated a role for iron acquisition in *C. jejuni* intracellular survival (Naikare et al., 2006). This therefore piqued our interest in identifying a role for a Tlp involved in chemotaxis toward iron, in *C. jejuni* survival within host cells. The results of our study showed that the deletion of *tlp2* increased intracellular recovery of *C. jejuni*. This was in contrary to our belief that deletion of *tlp2* would decrease the survival of *C. jejuni* in host cells, due to the decreased chemotaxis toward iron. It must however, be noted that intracellular *C. jejuni* undergo a metabolic reprogramming which affects their survival within epithelial cells (Svensson et al., 2009; Liu et al., 2012). The increased intracellular survival in the Δ*tlp2* mutant may indicate a dysregulation of cellular process which warrants further investigation.

The role of *tlp2* in tissue specific colonization of the chicken gastrointestinal tract was investigated. Mutation in *tlp2* resulted in a colonization defect in the cecum, with a more profound reduction seen in the duodenum and jejunum. Catabolism of amino acids such as aspartate and serine are essential for *C. jejuni* colonization of the avian gut (Guccione et al., 2008), as reflected by the *tlp1* mutant (aspartate chemoreceptor), which was severely impaired in colonization of the chicken ceca (Hartley-Tassell et al., 2010). The *tlp2* mutant demonstrated a decreased chemotaxis toward aspartate, which might explain the reduced colonization. The utilization of glutamine, glutathione and asparagine in *C. jejuni* 81–176 is associated to tissue-specific colonization of the murine intestine (Hofreuter et al., 2008). However, it is not known if the ability to metabolize these nutrients also supports tissue specific colonization in the chicken intestinal tract. Additionally, chemotaxis toward pyruvate and fumarate mediated by Tlp9 represents energy taxis in *C. jejuni*. Energy taxis is an essential driving force for *C. jejuni* for establishment during colonization of the host (Vegge et al., 2009). The chicken cecum represents an iron and phosphate limiting environment for *C. jejuni* and iron acquisition is known to be essential for *C. jejuni* colonization of the chicken (Naikare et al., 2006). It is not surprising to see that Δ*tlp2* mutant, defective in chemotaxis toward Pi and iron, is also defective in colonization of the chicken cecum, duodenum, and jejunum. These findings clearly indicate that *tlp2* contributes to *C jejuni* interaction with host cells, which is an important determinant for *C jejuni* pathogenesis and colonization of the chicken gastrointestinal tract.

In summary, the present study identifies a role for *tlp2* in *C. jejuni* chemotaxis, stress survival and colonization of the chicken gastrointestinal tract. Further, our findings indicate that iron regulates *tlp2.* The *tlp2* mutant was also defective in chemotaxis to Pi and showed increased PhoX activity. This suggests a possible cross-talk between iron and phosphate regulatory pathways, which needs further investigation. In addition, the increased PhoX activity in the presence of iron seen in *C. jejuni* indicates that iron may reduce the bioavailability of phosphate. Our findings in this study suggest a basis for future biochemical characterization of PhoX in *C. jejuni*.

## Ethics Statement

Animal experiments were conducted according to the guidelines of the Association for Assessment and Accreditation of Laboratory Animal Care International (AAALAC). The animal studies were approved by the Institutional Animal Care and Use Committee (IACUC), the Ohio State University. Chickens were housed at the Food Animal Health Research Program Animal Care Facility, which is fully accredited by AAALAC and the animals were supervised by a senior veterinarian. Infectious agents were administered using manual restraint for less than one minute to minimize distress. Before necropsy, chickens were euthanized by carbon dioxide inhalation. This method is consistent with the recommendations of the panel on euthanasia of the American Veterinary Medical Association and by the Ohio State University Institutional Laboratory Animal Care and Use Committee.

## Author Contributions

GR and KC designed the experiments. KC, SH, BJ, and SR performed the experiments and collected the data. KC, GR and VS analyzed the data. KC, GR, VS, and BJ wrote the paper.

## Conflict of Interest Statement

The authors declare that the research was conducted in the absence of any commercial or financial relationships that could be construed as a potential conflict of interest. The reviewer YK and handling Editor declared their shared affiliation.

## References

[B1] Anantharaman V. Aravind L. (2000). Cache - a signaling domain common to animal Ca(2 + )-channel subunits and a class of prokaryotic chemotaxis receptors. *Trends Biochem. Sci.* 25 535–537. 10.1016/S0968-0004(00)01672-8 11084361

[B2] Bencharit S. Ward M. J. (2005). Chemotactic responses to metals and anaerobic electron acceptors in Shewanella oneidensis MR-1. *J. Bacteriol.* 187 5049–5053. 10.1128/JB.187.14.5049-5053.2005 15995227 PMC1169517

[B3] Braun V. Hantke K. (2003). “Mechanisms of bacterial iron transport,” in *Microbial Transport System*, ed. Winkelmann G. (Weinheim: Wiley-VCH Verlag GmBH & Co), 289–311.

[B4] Butcher J. Sarvan S. Brunzelle J. S. Couture J. F. Stintzi A. (2012). Structure and regulon of *Campylobacter jejuni* ferric uptake regulator Fur define apo-Fur regulation. *Proc. Natl. Acad. Sci. U.S.A.* 109 10047–10052. 10.1073/pnas.1118321109 22665794 PMC3382491

[B5] Candon H. L. Allan B. J. Fraley C. D. Gaynor E. C. (2007). Polyphosphate kinase 1 is a pathogenesis determinant in Campylobacter jejuni. *J. Bacteriol.* 189 8099–8108. 10.1128/JB.01037-1037 17827292 PMC2168705

[B6] CDC. (2013). “Incidence and trends of infection with pathogens transmitted commonly through food — foodborne diseases active surveillance network, 10 u.s. sites, 1996–2012,” in *Morbidity and Mortality Weekly Report*, ed. Charlotte K. (Washington, DC: U.S. Government PrintingOffice). PMC4604974 23594684

[B7] Cerda O. Rivas A. Toledo H. (2003). Helicobacter pylori strain ATCC700392 encodes a methyl-accepting chemotaxis receptor protein (MCP) for arginine and sodium bicarbonate. *FEMS Microbiol. Lett.* 224 175–181. 10.1016/S0378-1097(03)00423-3 12892880

[B8] Cerda O. A. Nunez-Villena F. Soto S. E. Ugalde J. M. Lopez-Solis R. Toledo H. (2011). tlpA gene expression is required for arginine and bicarbonate chemotaxis in helicobacter pylori. *Biol. Res.* 44 277–282. /S0716-97602011000300009 22688915

[B9] Chandrashekhar K. Gangaiah D. Pina-Mimbela R. Kassem I. I. Jeon B. H. Rajashekara G. (2015). Transducer like proteins of *Campylobacter jejuni* 81-176: role in chemotaxis and colonization of the chicken gastrointestinal tract. *Front. Cell. Infect. Microbiol.* 5:46. 10.3389/fcimb.2015.00046 26075188 PMC4444964

[B10] Chandrashekhar K. Kassem I. I. Rajashekara G. (2017). *Campylobacter jejuni* transducer like proteins: chemotaxis and beyond. *Gut Microbes* 8 323–334. 10.1080/19490976.2017.1279380 28080213 PMC5570417

[B11] Childers S. E. Ciufo S. Lovley D. R. (2002). Geobacter metallireducens accesses insoluble Fe(III) oxide by chemotaxis. *Nature* 416 767–769. 10.1038/416767a. 11961561

[B12] Day C. J. Hartley-Tassell L. E. Shewell L. K. King R. M. Tram G. Day S. K. (2012). Variation of chemosensory receptor content of *Campylobacter jejuni* strains and modulation of receptor gene expression under different in vivo and in vitro growth conditions. *BMC Microbiol.* 12:128. 10.1186/1471-2180-12-128 22747654 PMC3461409

[B13] Drozd M. Chandrashekhar K. Rajashekara G. (2014). polyphosphate-mediated modulation of *Campylobacter jejuni* biofilm growth and stability. *Virulence* 5 680–690. 10.4161/viru.34348 25127528 PMC4139409

[B14] Drozd M. Gangaiah D. Liu Z. Rajashekara G. (2011). Contribution of TAT system translocated PhoX to *Campylobacter jejuni* phosphate metabolism and resilience to environmental stresses. *PLoS One* 6:e26336. 10.1371/journal.pone.0026336 22028859 PMC3197622

[B15] Ernst F. D. Bereswill S. Waidner B. Stoof J. Mader U. Kusters J. G. (2005). Transcriptional profiling of Helicobacter pylori Fur- and iron-regulated gene expression. *Microbiology* 151(Pt 2), 533–546. 10.1099/mic.0.27404-27400 15699202

[B16] Food and Drug Administration and HHS. (2014). Establishing a list of qualifying pathogens under the food and drug administration safety and innovation act: final rule. *Fed. Regist.* 79 32464–32481. 24908687

[B17] Gangaiah D. Kassem I. I Liu Z. Rajashekara G. (2009). Importance of polyphosphate kinase 1 for *Campylobacter jejuni* viable-but-nonculturable cell formation, natural transformation, and antimicrobial resistance. *Appl. Environ. Microbiol.* 75 7838–7849. 10.1128/AEM.01603-1609 19837830 PMC2794102

[B18] Gangaiah D. Liu Z. Arcos J. Kassem I. I Sanad Y. Torrelles J. B. (2010). Polyphosphate kinase 2: a novel determinant of stress responses and pathogenesis in *Campylobacter jejuni*. *PLoS One* 5:e12142. 10.1371/journal.pone.0012142 20808906 PMC2923150

[B19] Grabowska A. D. Wandel M. P. Lasica A. M. Nesteruk M. Roszczenko P. Wyszynska A. (2011). *Campylobacter jejuni* dsb gene expression is regulated by iron in a Fur-dependent manner and by a translational coupling mechanism. *BMC Microbiol.* 11:166. 10.1186/1471-2180-11-166 21787430 PMC3167755

[B20] Guccione E. Leon-Kempis Mdel R. Pearson B. M. Hitchin E. Mulholland F. van Diemen P. M. (2008). Amino acid-dependent growth of *Campylobacter jejuni*: key roles for aspartase (AspA) under microaerobic and oxygen-limited conditions and identification of AspB (Cj0762), essential for growth on glutamate. *Mol. Microbiol.* 69 77–93. 10.1111/j.1365-2958.2008.06263.x 18433445

[B21] Harris H. W. El-Naggar M. Y. Bretschger O. Ward M. J. Romine M. F. Obraztsova A. Y. (2010). Electrokinesis is a microbial behavior that requires extracellular electron transport. *Proc. Natl. Acad. Sci. U.S.A.* 107 326–331. 10.1073/pnas.0907468107 20018675 PMC2806741

[B22] Hartley-Tassell L. E. Shewell L. K. Day C. J. Wilson J. C. Sandhu R. Ketley J. M. (2010). Identification and characterization of the aspartate chemosensory receptor of *Campylobacter jejuni*. *Mol. Microbiol.* 75 710–730. 10.1111/j.1365-2958.2009.07010.x 20025667

[B23] Hendrixson D. R. DiRita V. J. (2004). Identification of *Campylobacter jejuni* genes involved in commensal colonization of the chick gastrointestinal tract. *Mol. Microbiol.* 52 471–484. 10.1111/j.1365-2958.2004.03988.x 15066034

[B24] Hermans D. Pasmans F. Messens W. Martel A. Van Immerseel F. Rasschaert G. (2012). Poultry as a host for the zoonotic pathogen *Campylobacter jejuni*. *Vector Borne Zoonotic Dis.* 12 89–98. 10.1089/vbz.2011.0676 22133236

[B25] Hermans D. Van Deun K. Martel A. Van Immerseel F. Messens W. Heyndrickx M. (2011). Colonization factors of *Campylobacter jejuni* in the chicken gut. *Vet. Res.* 42:82. 10.1186/1297-9716-42-82 21714866 PMC3156733

[B26] Hofreuter D. Novik V. Galan J. E. (2008). Metabolic diversity in *Campylobacter jejuni* enhances specific tissue colonization. *Cell Host Microbe* 4 425–433. 10.1016/j.chom.2008.10.002 18996343

[B27] Holmes K. Mulholland F. Pearson B. M. Pin C. McNicholl-Kennedy J. Ketley J. M. (2005). *Campylobacter jejuni* gene expression in response to iron limitation and the role of fur. *Microbiology* 151(Pt 1), 243–257. 10.1099/mic.0.27412-27410 15632442

[B28] Hugdahl M. B. Beery J. T. Doyle M. P. (1988). Chemotactic behavior of *Campylobacter jejuni*. *Infect. Immun.* 56 1560–1566. 3372020 10.1128/iai.56.6.1560-1566.1988 PMC259436

[B29] Jeon B. Muraoka W. Sahin O. Zhang Q. (2008). Role of cj1211 in natural transformation and transfer of antibiotic resistance determinants in *Campylobacter jejuni*. *Antimicrob. Agents Chemother.* 52 2699–2708. 10.1128/aac.01607-1607 18505858 PMC2493120

[B30] Kassem I. I. Khatri M. Esseili M. A. Sanad Y. M. Saif Y. M. Olson J. W. (2012). Respiratory proteins contribute differentially to *Campylobacter jejuni’s* survival and in vitro interaction with hosts’ intestinal cells. *BMC Microbiol.* 12:258. 10.1186/1471-2180-12-258 23148765 PMC3541246

[B31] Kim M. Hwang S. Ryu S. Jeon B. (2011). Regulation of perR expression by iron and PerR in *Campylobacter jejuni*. *J. Bacteriol.* 193 6171–6178. 10.1128/JB.05493-5411 21908670 PMC3209207

[B32] Korlath J. A. Osterholm M. T. Judy L. A. Forfang J. C. Robinson R. A. (1985). A point-source outbreak of campylobacteriosis associated with consumption of raw milk. *J. Infect. Dis.* 152 592–596. 10.1093/infdis/152.3.592 4031557

[B33] Korolik V. (2010). Aspartate chemosensory receptor signalling in *Campylobacter jejuni*. *Virulence* 1 414–417. 10.4161/viru.1.5.12735 21178481

[B34] Kusaka K. Shibata K. Kuroda A. Kato J. Ohtake H. (1997). Isolation and characterization of Enterobacter cloacae mutants which are defective in chemotaxis toward inorganic phosphate. *J. Bacteriol.* 179 6192–6195. 10.1128/jb.179.19.6192-6195.1997 9324271 PMC179527

[B35] Lertsethtakarn P. Ottemann K. M. Hendrixson D. R. (2011). Motility and chemotaxis in *Campylobacter* and *Helicobacter*. *Annu. Rev. Microbiol.* 65 389–410. 10.1146/annurev-micro-090110-102908 21939377 PMC6238628

[B36] Lill R. (2009). Function and biogenesis of iron-sulphur proteins. *Nature* 460 831–838. 10.1038/nature08301 19675643

[B37] Liu X. Gao B. Novik V. Galan J. E. (2012). Quantitative proteomics of intracellular *Campylobacter jejuni* reveals metabolic reprogramming. *PLoS Pathog* 8:e1002562. 10.1371/journal.ppat.1002562 22412372 PMC3297583

[B38] Livak K. J. Schmittgen T. D. (2001). Analysis of relative gene expression data using real-time quantitative PCR and the 2(-Delta Delta C(T)) method. *Methods* 25 402–408. 10.1006/meth.2001.1262 11846609

[B39] Marchant J. Wren B. Ketley J. (2002). Exploiting genome sequence: predictions for mechanisms of Campylobacter chemotaxis. *Trends Microbiol.* 10 155–159. 10.1016/S0966-842X(02)02323-5 11912013

[B40] Mazumder R. Phelps T. J. Krieg N. R. Benoit R. E. (1999). Determining chemotactic responses by two subsurface microaerophiles using a simplified capillary assay method. *J. Microbiol. Methods* 37 255–263. 10.1016/S0167-7012(99)00072-X 10480269

[B41] Miller W. G. Bates A. H. Horn S. T. Brandl M. T. Wachtel M. R. Mandrell R. E. (2000). Detection on surfaces and in Caco-2 cells of *Campylobacter jejuni* cells transformed with new gfp, yfp, and cfp marker plasmids. *Appl. Environ. Microbiol.* 66 5426–5436. 10.1128/AEM.66.12.5426-5436.2000 11097924 PMC92478

[B42] Naikare H. Palyada K. Panciera R. Marlow D. Stintzi A. (2006). Major role for FeoB in *Campylobacter jejuni* ferrous iron acquisition, gut colonization, and intracellular survival. *Infect. Immun.* 74 5433–5444. 10.1128/IAI.00052-56 16988218 PMC1594910

[B43] Nishiyama S. Suzuki D. Itoh Y. Suzuki K. Tajima H. Hyakutake A. (2012). Mlp24 (McpX) of *Vibrio cholerae* implicated in pathogenicity functions as a chemoreceptor for multiple amino acids. *Infect. Immun.* 80 3170–3178. 10.1128/IAI.00039-12 22753378 PMC3418727

[B44] Rahman H. King R. M. Shewell L. K. Semchenko E. A. Hartley-Tassell L. E. Wilson J. C. (2014). Characterisation of a multi-ligand binding chemoreceptor ccml (tlp3) of *Campylobacter jejuni*. *PLoS Pathog* 10:e1003822. 10.1371/journal.ppat.1003822 24391495 PMC3879368

[B45] Rajashekara G. Drozd M. Gangaiah D. Jeon B. Liu Z. Zhang Q. (2009). Functional characterization of the twin-arginine translocation system in *Campylobacter jejuni*. *Foodborne Pathog Dis.* 6 935–945. 10.1089/fpd.2009.0298 19799526

[B46] Reuter M. van Vliet A. H. (2013). Signal balancing by the CetABC and CetZ chemoreceptors controls energy taxis in *Campylobacter jejuni*. *PLoS One* 8:e54390. 10.1371/journal.pone.0054390 23382896 PMC3558505

[B47] Sambrook J. Fritsch E. F. Maniatis T. (1989). Molecular cloning: a laboratory manual. (Cold Spring Harbor, NY: Cold Spring Harbor Laboratory Press).

[B48] Sanders L. Andermann T. M. Ottemann K. M. (2013). A supplemented soft agar chemotaxis assay demonstrates the Helicobacter pylori chemotactic response to zinc and nickel. *Microbiology* 159(Pt 1), 46–57. 10.1099/mic.0.062877-62870 23139399 PMC3542728

[B49] Schultz J. Milpetz F. Bork P. Ponting C. P. (1998). SMART, a simple modular architecture research tool: identification of signaling domains. *Proc. Natl. Acad. Sci. U.S.A.* 95 5857–5864. 10.1073/pnas.95.11.5857 9600884 PMC34487

[B50] Svensson S. L. Davis L. M. MacKichan J. K. Allan B. J. Pajaniappan M. Thompson S. A. (2009). The CprS sensor kinase of the zoonotic pathogen *Campylobacter jejuni* influences biofilm formation and is required for optimal chick colonization. *Mol. Microbiol.* 71 253–272. 10.1111/j.1365-2958.2008.06534.x 19017270 PMC2771394

[B51] Tareen A. M. Dasti J. I. Zautner A. E. Gross U. Lugert R. (2010). *Campylobacter jejuni* proteins Cj0952c and Cj0951c affect chemotactic behaviour towards formic acid and are important for invasion of host cells. *Microbiology* 156(Pt 10), 3123–3135. 10.1099/mic.0.039438-39430 20656782

[B52] Tasneem A. Iyer L. M. Jakobsson E. Aravind L. (2005). Identification of the prokaryotic ligand-gated ion channels and their implications for the mechanisms and origins of animal Cys-loop ion channels. *Genome Biol.* 6:R4. 10.1186/gb-2004-6-1-r4 15642096 PMC549065

[B53] van Vliet A. H. Wooldridge K. G. Ketley J. M. (1998). Iron-responsive gene regulation in a *Campylobacter jejuni* fur mutant. *J. Bacteriol.* 180 5291–5298. 9765558 10.1128/jb.180.20.5291-5298.1998 PMC107575

[B54] Vegge C. S. Brondsted L. Li Y. P. Bang D. D. Ingmer H. (2009). Energy taxis drives *Campylobacter jejuni* toward the most favorable conditions for growth. *Appl. Environ. Microbiol.* 75 5308–5314. 10.1128/AEM.00287-289 19542337 PMC2725471

[B55] Watson R. O. Galan J. E. (2008). *Campylobacter jejuni* survives within epithelial cells by avoiding delivery to lysosomes. *PLoS Pathog* 4:e14. 10.1371/journal.ppat.0040014 18225954 PMC2323279

[B56] Wosten M. M. Boeve M. Koot M. G. van Nuenen A. C. van der Zeijst B. A. (1998). Identification of *Campylobacter jejuni* promoter sequences. *J. Bacteriol.* 180 594–599. 9457862 10.1128/jb.180.3.594-599.1998 PMC106926

[B57] Wosten M. M. Parker C. T. van Mourik A. Guilhabert M. R. van Dijk L. van Putten J. P. (2006). The *Campylobacter jejuni* PhosS/PhosR operon represents a non-classical phosphate-sensitive two-component system. *Mol. Microbiol.* 62 278–291. 10.1111/j.1365-2958.2006.05372.x 16956379

[B58] Wu H. Kato J. Kuroda A. Ikeda T. Takiguchi N. Ohtake H. (2000). Identification and characterization of two chemotactic transducers for inorganic phosphate in *Pseudomonas aeruginosa*. *J. Bacteriol.* 182 3400–3404. 10.1128/JB.182.12.3400-3404.2000 10852870 PMC101905

[B59] Yao R. Alm R. A. Trust T. J. Guerry P. (1993). Construction of new *Campylobacter* cloning vectors and a new mutational cat cassette. *Gene* 130 127–130. 10.1016/0378-1119(93)90355-7 8344519

[B60] Yao R. Burr D. H. Doig P. Trust T. J. Niu H. Guerry P. (1994). Isolation of motile and non-motile insertional mutants of *Campylobacter jejuni*: the role of motility in adherence and invasion of eukaryotic cells. *Mol. Microbiol.* 14 883–893. 10.1111/j.1365-2958.1994.tb01324.x 7715450

[B61] Yao R. Burr Donald H. Guerry P. (1997). CheY-mediated modulation of *Campylobacter jejuni* virulence. *Mol. Microbiol.* 23 1021–1031. 10.1046/j.1365-2958.1997.2861650.x 9076738

[B62] Yong S. C. Roversi P. Lillington J. Rodriguez F. Krehenbrink M. Zeldin O. B. (2014). A complex iron-calcium cofactor catalyzing phosphotransfer chemistry. *Science* 345 1170–1173. 10.1126/science.1254237 25190793 PMC4175392

[B63] Young K. T. Davis L. M. Dirita V. J. (2007). ‘Campylobacter jejuni: molecular biology and pathogenesis. *Nat. Rev. Microbiol.* 5 665–679. 10.1038/nrmicro1718 17703225

