# Supplementary material for: Transducer-Like Protein in Campylobacter jejuni With a Role in Mediating Chemotaxis to Iron and Phosphate
Source: Front Microbiol. 2018 Nov 16;9:2674. doi: 10.3389/fmicb.2018.02674 (PMC6250842; doi:10.3389/fmicb.2018.02674)
Supplement: Supplementary file 1 [file Data_Sheet_1.PDF]

Figure S1

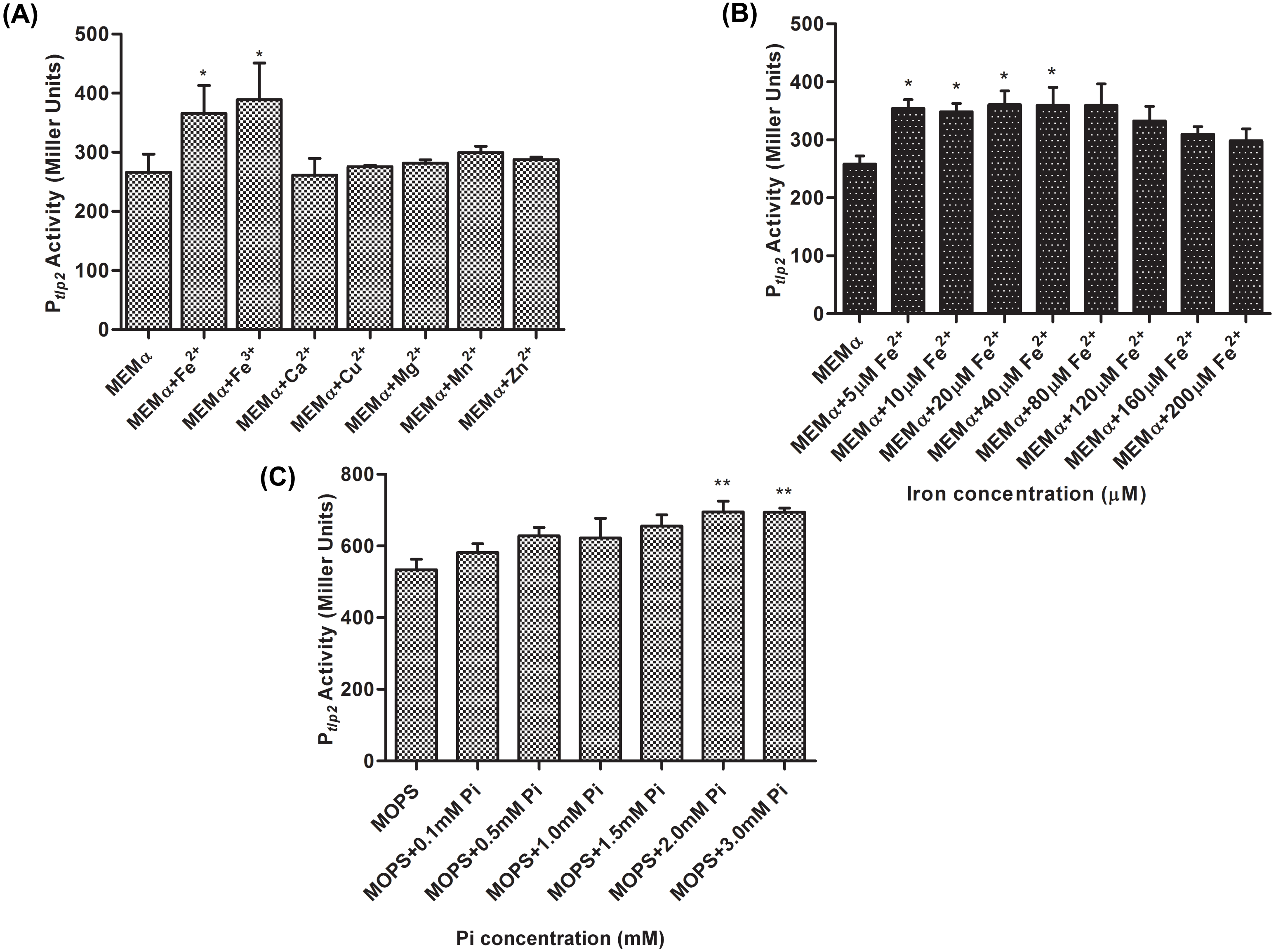

Figure S1. **(A)** P_{tlp2}-*lacZ* fusion assays in the presence of different metals/cations based on previously published concentrations (Kim et al., 2011) added to MEMα. (**B**) P_{tlp2}-*lacZ* fusion dose response assays in the presence of different concentrations of $FeSO_4$ added to MEMα. **(C)** P_{tlp2}-*lacZ* fusion dose response assays in the presence of different concentrations of Pi (Lithium Potassium Acetyl Phosphate; used as phosphate source) ranging from 0.1mM to 3mM. The cells were incubated for 8 h before carrying out the assay. The results show the means and standard deviations of three independent experiments. * $P < 0.05$. ** $P < 0.01$

Figure S2

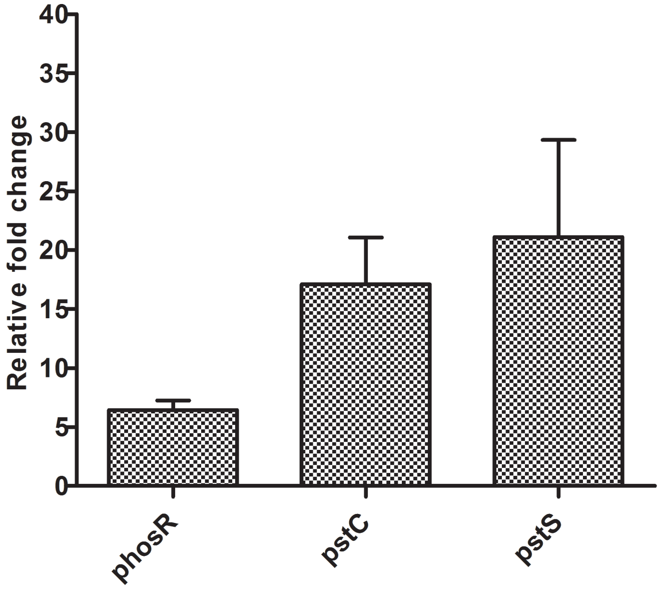

Figure S2. The relative change in the expression of *C. jejuni* phosphate uptake genes; *phosR, pstC* and *pstS* in the Δ*tlp2* mutant by (q)RT-PCR. Strains were grown in MEM-α (minimal media) until mid-log phase and total RNA was extracted. The relative fold change in expression was calculated by $2^{-\Delta\Delta CT.}$
